# Sickle cell detection using a smartphone

**DOI:** 10.1038/srep15022

**Published:** 2015-10-22

**Authors:** S. M. Knowlton, I. Sencan, Y. Aytar, J. Khoory, M. M. Heeney, I. C. Ghiran, S. Tasoglu

**Affiliations:** 1 Department of Biomedical Engineering, University of Connecticut, 260 Glenbrook Road, Storrs, CT 06269; 2 Department of Diagnostic Radiology, Yale School of Medicine, New Haven, CT 06520-8043; 3 Computer Science and Artificial Intelligence Laboratory, Massachusetts Institute of Technology, Vassar Street Cambridge, MA 02139; 4 Department of Medicine, Beth Israel Deaconess Medical Center, Harvard Medical School, Boston MA 02115; 5 Sickle Cell Program, Dana-Farber/Boston Children’s Cancer and Blood Disorders Center, Harvard Medical School, Boston MA 02115; 6 Department of Mechanical Engineering, University of Connecticut, Storrs, CT 06269.

## Abstract

Sickle cell disease affects 25% of people living in Central and West Africa and, if left undiagnosed, can cause life threatening “silent” strokes and lifelong damage. However, ubiquitous testing procedures have yet to be implemented in these areas, necessitating a simple, rapid, and accurate testing platform to diagnose sickle cell disease. Here, we present a label-free, sensitive, and specific testing platform using only a small blood sample (<1 μl) based on the higher density of sickle red blood cells under deoxygenated conditions. Testing is performed with a lightweight and compact 3D-printed attachment installed on a commercial smartphone. This attachment includes an LED to illuminate the sample, an optical lens to magnify the image, and two permanent magnets for magnetic levitation of red blood cells. The sample is suspended in a paramagnetic medium with sodium metabisulfite and loaded in a microcapillary tube that is inserted between the magnets. Red blood cells are levitated in the magnetic field based on equilibrium between the magnetic and buoyancy forces acting on the cells. Using this approach, we were able to distinguish between the levitation patterns of sickle versus control red blood cells based on their degree of confinement.

In 2006, the World Health Organization (WHO) identified sickle cell disease (SCD) as a public health issue^1^, affecting around 25% of people in Central and West Africa and 70,000–100,000 people in the United States^2^. This hereditary disease is caused by a mutation in the beta globin gene which gives rise to an abnormal form of hemoglobin, called hemoglobin S^3^. In the deoxygenated T-state, hemoglobin S aggregates into long, straight fibers which deform the cell and cause it to take on the iconic sickle shape^4^. The functional consequences of hemoglobin aggregation involve inhibited blood flow, causing pain, organ damage, increased risk of stroke, infection, and other complications. Sickle cell anemia (SS genotype) is the most common and most severe form of sickle cell disease, due to a person inheriting two hemoglobin S genes, one from each parent. Others can carry only one hemoglobin S gene, which is considered sickle cell trait (AS genotype). Other forms of the disease are caused by inheriting another mutant form of the hemoglobin gene in addition to hemoglobin S^5^. Red blood cells (RBCs) are necessary for carrying oxygen and removing carbon dioxide through the blood stream. In a person without sickle cell disease, these cells survive for around 120 days and are replaced by new cells synthesized by bone marrow. However, mutant cells survive only 10 to 20 days, resulting in a hemolytic anemia characterized by a decrease in the number of circulating RBCs and total hemoglobin^3^.

The only known cure for sickle cell disease is a stem cell or bone marrow transplant; other treatments focus on managing the symptoms and complications. The median life expectancy for those with sickle cell anemia is 40 to 50 years^6^. In light of the impact of this disease, the WHO has called for design and implementation of programs for preventing and managing sickle cell anemia, including surveillance and screening programs, in countries where sickle cell anemia is a public health problem^1^. As of January 2006, all newborns in the United States are screened for sickle cell disease using a blood test on filter paper. However, confirmatory testing requires hemoglobin electrophoresis, isoelectric focusing, or high-performance liquid chromatography, which tend to be expensive and require specialized equipment and training^7^. For implementation in developing countries where the disease is prevalent, diagnostic devices and implementation programs must take into account socioeconomic, health system, and cultural contexts, thus necessitating a low cost, compact, and user friendly diagnostic device with sensitive and specific detection capabilities for sickle cell disease.

The use of smartphones for medical diagnostics offers accessibility, versatility, improved portability compared to larger instruments, and the potential to transmit medical results acquired in remote locations for epidemiological analysis. A myriad of engineered attachments and applications have been developed for smartphones in order to diagnose a range of medical conditions and track data for patient medical records and epidemiological studies. A wearable smartphone based platform was developed to collect electrocardiography signals and analyze them to diagnose cardiac arrhythmia^8^. A built-in smartphone camera and flash has been used to collect a pulsatile photoplethysmogram signal and an application was implemented to analyze the signal and diagnose atrial fibrillation^9^. Urine albumin testing using fluorescent assays within a smartphone attachment has been developed for early detection of kidney disease, diabetes, hypertension, and cardiovascular disease^10^. Other diagnostic platforms have been developed using a smartphone camera to measure red and white blood cell density and hemoglobin concentration from blood samples^11^, detect *Escherichia coli*^12^, and test for food allergens^13^. A rapid-diagnostic-test reader platform has also been developed which can test for a range of analytes of interest, including malaria, tuberculosis, and human immunodeficiency virus using various lateral flow immuno-chromatographic assays; results are then uploaded to a central server with geotagging data to facilitate tracking of epidemics on a global scale^14^. A smartphone camera has also been adapted to serve as a flow cytometer using an optofluidic fluorescent imaging platform^15^ and as a spectrophotometer for Enzyme Linked Immunosorbent Assays (ELISA) and colorimetric assays in the field^16^. Smartphone based microscopy has recently become an area of rapid innovation with the development of a phone-mounted light microscope^17^, a lens-free holographic microscopy platform^18^, a multi-frame imaging technique using a fiber optic array for high-density samples^19^, and a fluorescence microscopy platform capable of imaging fluorescence-tagged nanoparticles and viruses for multiple diagnostic applications^20^.

Current point of care diagnostic techniques for sickle cell disease include solubility tests based on the aggregation of hemoglobin S from lysed cells into an insoluble nematic liquid crystal^21^. Non-electrolyte haemolysis has also been proposed to diagnose sickle cell disease based on the altered properties of the sickle cell membrane due to hemoglobin aggregation^22^. Paper-based testing is based on chromatography using filter paper and analyzing the blood stain pattern formed^23^,^24^,^25^. Recently, density-based diagnostic testing has been presented as an alternative to the microscopy-based identification of cells with the characteristic sickle shape or other methods relying on detection of hemoglobin S^26^. Density-based detection offers greater sensitivity to different types of sickle cell disease. A recently developed method for density-based sickle cell disease diagnostics involves separation of blood cells by centrifugation in aqueous multiphase polymers^27^. More recently, magnetic levitation has been applied to detect density differences in cells due to permanent or transient changes in the RBCs’ magnetic and density signatures^28^.

Magnetic levitation is able to distinguish between control RBCs and relatively high density SS RBCs^26^, which is observable as a change in the levitation of the cells within a magnetic field^29^. The densities of SS RBCs vary, with most of them sharing the same density as most control RBCs of the same circulation age, but a large percentage of them having both the hallmark elongated “cigar-like or spindle-like” permanent shape change and a significantly higher density even when fully oxygenated. Several subpopulations of SS RBCs have been identified which exhibit a wide range of increases in density: reticulocytes, discocytes, dense discocytes, and irreversibly sickled cells^30^. Sodium metabisulfite is used as an oxygen scavenger to induce the deoxygenated form of hemoglobin that triggers a water-exclusion response of the cell. The mechanism involved in density increase starts through a Ca^2+^-dependent activation of Ca^2+^-sensitive K channels (IK1s or Gardos channels) in the cell membranes, which in turn promotes loss of potassium chloride (KCl) and water at a rate limited by the Cl^−^ permeability of the RBC membrane. Control RBCs can overcome this effect, recovering their initial density^31^. However, under these conditions, SS RBCs undergo an increase in density. This density change in RBCs is observable using this magnetic levitation platform as varying degrees of decreased levitation height of SS RBCs compared to control samples.

Here, we develop an alternative portable density-based diagnostic technique which requires only minimal volumes of blood and does not require the use of centrifugation equipment^28^. Use of magnetic levitation eliminates the need for a microscope to observe the sickle shape directly or centrifugation to separate cells based on density, but rather allows the use of smartphone imaging to observe, at a lower image resolution, the levitation height and degree of confinement of SS cells that underwent sodium metabisulfite-induced dehydration compared to the levitation of control cells. We present a 3D-printed magnetic levitation platform, termed Sickle Cell Tester, which is compatible with a Samsung Galaxy S4 smartphone^32^. The device confines RBCs in a microcapillary via magnetic levitation, captures images of the RBCs levitating in the magnetic field using the built in smartphone camera and an optical lens, and automatically analyses the RBC distribution with respect to the capillary edges using a custom-developed Android application running on the same smartphone. This platform does not require an internet connection and achieves label-free, sensitive, and specific detection of sickle cell disease, providing a useful point-of-care diagnosis tool.

## Results

### Sickle Cell Tester fabrication

**Figure 1:**
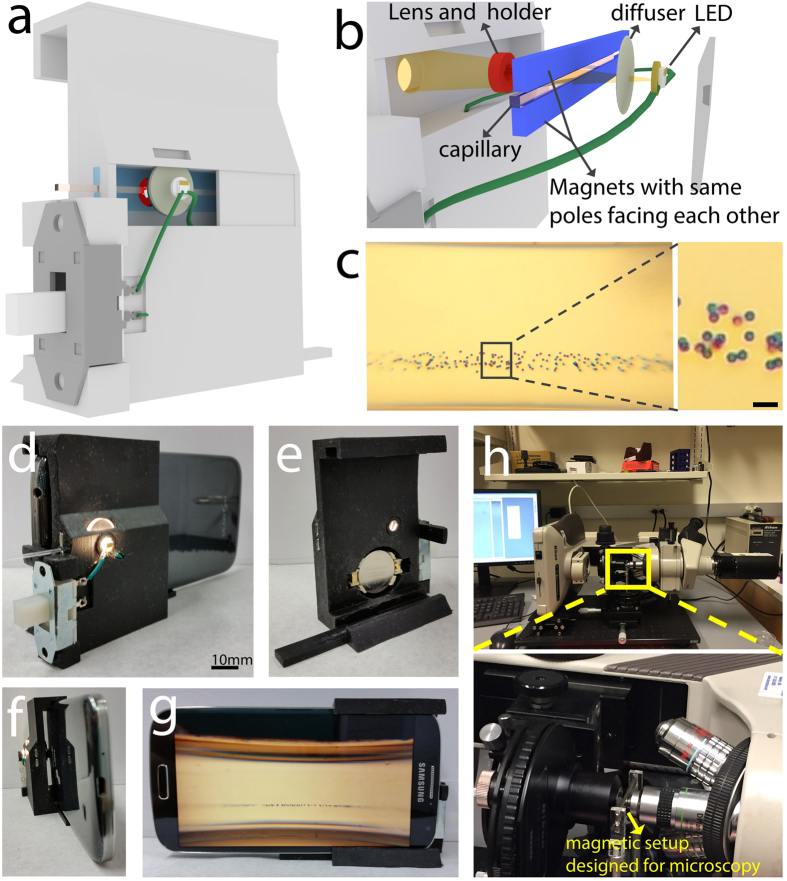
Magnetic levitation based platform (Sickle Cell Tester) installed on a smartphone and optical microscope for static separation of cells. (**a**) A schematic diagram of the lightweight smartphone attachment of the Sickle Cell Tester is illustrated. The smartphone attachment is composed of a 3D-printed holder, optical components (aspheric lens and holder), LED illumination, and two permanent magnets with the same poles facing each other. A capillary may be inserted between the magnets by the user. (**b**) The sample is illuminated with an LED through a ground glass diffuser. The image is magnified by the aspheric lens before it is captured by the built-in smartphone camera (not pictured). (**c**) Captured image of magnetic levitation of 10 μm polystyrene microspheres (scale bar is 25 μm). (**d**–**f**) Front, back, and side view images of the Sickle Cell Tester platform. (**g**) An image of levitating microspheres captured with the smartphone. (**h**) A light microscope laid on its side that allows imaging of levitating cells using an comparable two-magnet configuration to the one shown in (**a**).

The smartphone attachment is composed of a 3D printed holder containing: (i) optical components (an aspheric lens, 3D printed lens frame, diffuser, and LED), (ii) a battery and on/off switch to power an LED, and (iii) two permanent neodymium (NdFeB) magnets with like poles facing each other (Fig. 1a, Supplementary Figure 1)^32^. The attachment is designed to stabilize the smartphone upright on its side edge and can be easily modified for other smartphones. In the magnetic levitation component of the smartphone attachment, a capillary with a 1 mm × 1 mm square cross-section may be placed by the user between the two magnets. The smartphone camera captures images of the microcapillary through a 0.63 NA aspheric lens that is inserted between the magnetic setup and the smartphone camera lens to magnify the image (Fig. 1b). An LED and diffuser at the far side of the capillary surface are used to enhance imaging (Fig. 1b). Microcapillary tubes are disposable and easy to use for sample loading, as the sample is drawn into the capillary via capillary action. When RBCs are suspended in a biocompatible paramagnetic solution and exposed to the magnetic field, they are levitated and confined in the microcapillary (Fig. 1c), This process is dependent on their volumetric mass density and magnetic susceptibility; the platform takes advantage of the lower levitation height of objects with higher densities (in this case, SS RBCs relative to control RBCs), offering an easy yet powerful method for label-free visual quantification, especially for point-of-care settings (Fig. 1d–g). Additionally, we fabricated a separate capillary holder compatible with microscopy to stabilize magnets with the same poles facing each other for comparable magnetic levitation and imaging^28^. Representative images are shown in Fig. 1h to demonstrate the scale difference between Sickle Cell Tester and a similar process using a conventional optical microscope^28^, highlighting the relative compactness of the Sickle Cell Tester platform for imaging magnetically levitated cells (Fig. 1h).

### Characterization of magnetic levitation

**Figure 2:**
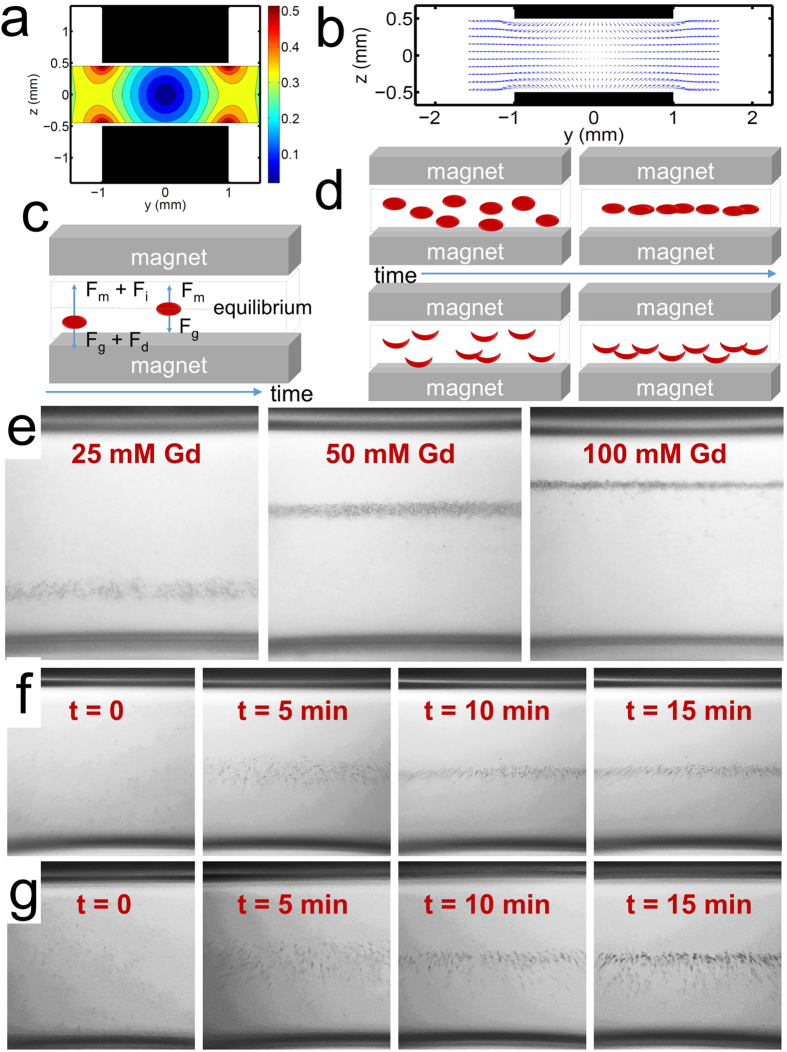
Theoretical and experimental magnetic levitation of RBCs. (**a**) Contour plot (**b**) magnetic field lines representing the magnetic field between the two magnets with like poles facing each other. The magnitude of the magnetic field is constrained between 0 T and 0.4 T. (**c**) Until the object reaches equilibrium, fluidic drag (F_d_), inertial (F_i_), buoyancy (F_g_), and magnetic forces (F_m_) act on the object. As the object gets closer to the equilibrium between F_g_ and F_m_, its migration velocity, and thus drag and inertial forces, become smaller. (**d**) Theoretical levitation of control RBCs (top) and SS RBCs (bottom), where some SS RBCs levitate at a lower height than control RBCs due to their higher density. (**e**) Levitation of RBCs in different concentrations of gadolinium solution, demonstrating the effect of the relative magnetic susceptibility on RBC levitation. Time-dependent confinement of (**f**) control and (**g**) SS RBCs toward equilibrium in a 50 mM Gd solution with 10 mM sodium metabisulfite.

The magnetic field strength formed by the two magnets with like poles facing each other approaches zero along the centerline of the microcapillary and increases toward the top and the bottom up to 0.4 T (Fig. 2a,b). Thus, the magnetic field exerts an upward force on a suspended diamagnetic object with a magnitude directly dependent on the location (height) of the object. The negative difference between the magnetic susceptibilities of an object with a small magnetic moment (*χ*_0_) and the paramagnetic suspending medium (*χ*_*medium*_) causes the object to move away from the stronger magnetic field near the magnets and toward the lower magnetic field at the centerline between the magnets. The buoyancy force, caused by gravity and density differences between the object and the medium, acts downward on the object. This equilibrium causes the object to levitate to a specific height at which the net force acting on it is equal to zero, which depends on both its buoyancy and magnetic susceptibility relative the medium (Fig. 2c). In this way, SS RBCs, some of which attain a higher density when exposed to sodium metabisulfite and thus experience an increased downward gravitational force, levitate at a lower height than control RBCs (Fig. 2d). The levitation height is also dependent on the concentration of gadolinium in the paramagnetic medium, where higher concentrations increase the magnetic force on the RBCs resulting in a levitation height closer to the centerline and narrower width of confinement (Fig. 2e). The time-dependent levitation of control and SS RBCs in the magnetic field is shown in Fig. 2f,g, respectively. Upon loading, RBCs were first homogeneously distributed in the media (t = 0) and gradually approached an equilibrium height. In a 50 mM gadolinium solution, the RBCs reached this stable levitation height after approximately 10 minutes, with minimal change seen in subsequent images (15 minutes). During the experiments, no adverse effects of the magnets on the screen resolution or any internal smartphone processes were observed.

### Android application image analysis

The captured images of the sample within the microcapillary are then digitally processed within 1 second using a custom-developed Android application running on the same smartphone. The application scans the image pixels in the x-direction and saves two arrays: **(i)** the average of the pixel intensities, and **(ii)** the gradients of pixel intensity changes in the x-direction. From these two arrays, the application recognizes the RBC confinement and evaluates a Gaussian fit to the pixel intensities in that region. The width of the confinement is approximated as four standard deviations (4σ) of this Gaussian fit. The application reports two outputs on the screen: **(i)** the y-location of RBC confinement (given as ‘mean’ at the bottom-right corner of the screen) and, (ii) the 4σ value of the fit (given as ‘std’ at the bottom-right corner of the screen).

**Figure 3:**
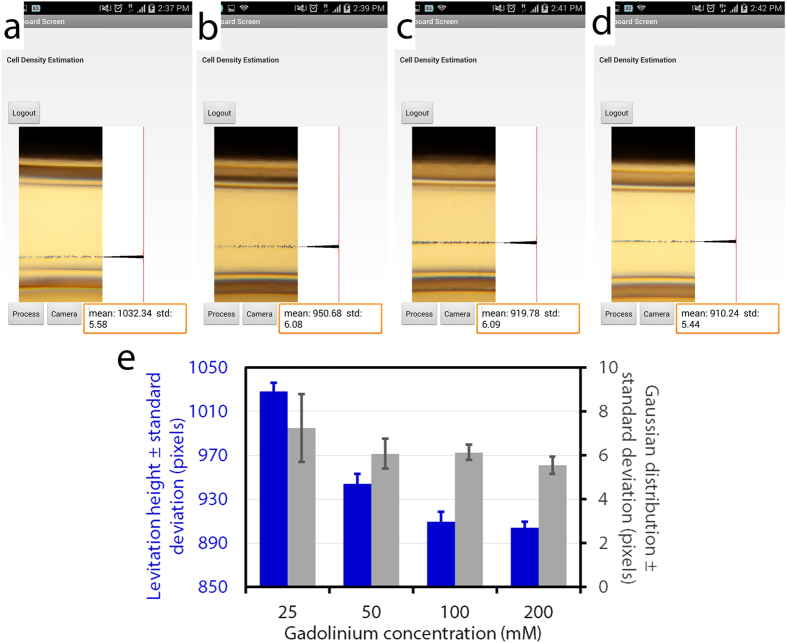
Cell levitation analysis application running on an Android phone is used to demonstrate the effect of gadolinium concentration on levitation height. (**a**–**d**) Images of levitating microspheres are acquired by the user and rapidly processed by the application to determine the levitation height (‘mean’) and confinement width (‘std’) of the microspheres in the sample. (**a**) 25 mM, (**b**) 50 mM, (**c**) 100 mM, and (**d**) 200 mM gadolinium solutions are tested. (**e**) The levitation height results (‘mean’, blue) show the varying levitation of microspheres in different concentrations of gadolinium (values are measured relative to the top magnet). The Gaussian distribution results (‘std’, gray) show that the width of confinement is fairly constant across all concentrations of gadolinium with a slight decrease in the width of confinement and a decrease in the variation between samples at higher concentrations of gadolinium. Error bars represent the standard deviation over 6 trials.

The effect of the concentration of gadolinium due to the change in the relative magnetic susceptibility and thus the upward magnetic force was quantified using the application. Polymer microspheres, which are also denser than the medium, were used as uniform-density micro-objects to represent RBCs. Higher gadolinium concentrations increased the magnetic force on the sample, thus increasing the rate at which the microspheres approach equilibrium and increasing the equilibrium height at which the microspheres levitated. The smartphone application was used to quantify this observation by measuring the levitation height in four different concentrations of gadolinium: 25 mM, 50 mM, 100 mM, and 200 mM (Fig. 3a–d), respectively. To demonstrate the repeatability of Sickle Cell Tester’s performance, ‘mean’ and ‘std’ results averaged over 6 trials for each case are shown in Fig. 3e. Standard deviations of the ‘mean’ and ‘std’ outputs are given as error bars.

### Magnetic levitation of control and SS RBCs

**Figure 4:**
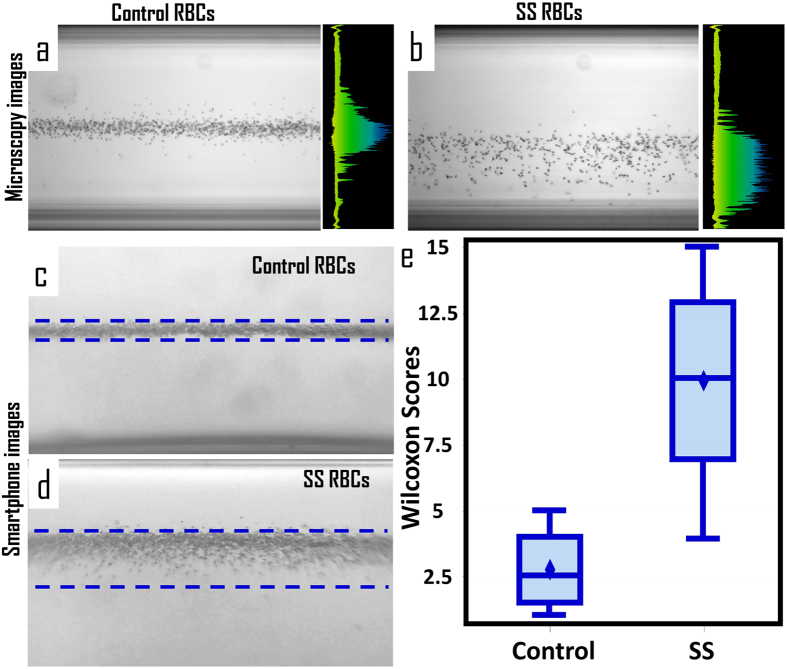
Identification of sickle cell disease using the magnetic levitation platform. Levitation of (**a**) control and (**b**) SS RBCs in 50 mM gadolinium solution with 10 mM sodium metabisulfite using a microscopy compatible-setup (Fig. 1h). Levitation of (**c**) control and (**d**) SS RBCs in 50 mM gadolinium solution with 10 mM sodium metabisulfite using the Sickle Cell Tester installed on a smartphone. (**e**) Distribution of Wilcoxon scores for confinement width of 4 control and 11 SS RBC samples analyzed using the Sickle Cell Tester. Results show a statistically significant difference between the groups according to a two-tailed non-parametric Mann-Whitney-Wilcoxon test with a t-approximation (α = 0.05). The full distribution of confinement widths are given in Supplementary Figure 2.

To test the effectiveness of the platform to detect sickle cell disease, blood samples from control donors and donors previously diagnosed with sickle cell disease were deoxygenated and dehydrated with 10 mM sodium metabisulfite dissolved in the paramagnetic gadolinium solution throughout the experiment to induce a density increase in SS cells. The paramagnetic solution used for all experiments presented here, Gadavist, is currently employed for MRI investigations in humans throughout the world. The gadolinium medium is non-toxic, iso-osmolar at the concentration used for imaging, and fully compatible with human blood cells^28^. When RBCs were suspended in the solution, placed in the magnetic field, and allowed to reach equilibrium (10 minutes), control cells (n = 4) were observed to levitate at a higher levitation height and within a narrower area of confinement than the SS cells (n = 11) in both the microscopy compatible setup (Fig. 4a,b) and Sickle Cell Tester (Fig. 4c,d). This is due to the increase in SS RBC density following sodium metabisulfite-induced deoxygenation and dehydration, which is significantly more pronounced in SS RBCs compared to control RBCs. The degree of confinement of the RBCs was calculated as the standard deviation of the RBC levitation heights; each result is the mean over 6 trials (Fig. 4e). A Mann-Whitney-Wilcoxon two-sided test was used to verify that the difference between distributions of the sickle cell group and the control group is significant (t approximation, n_1_ = 4, n_2_ = 11, p = 0.0181) (Fig. 4f). This quantifiable and repeatable difference indicates that this method of magnetic levitation of RBCs in sodium metabisulfite and gadolinium is a simple yet powerful binary measure for detection of sickle cell disease.

The SS RBCs, even in the absence of induced dehydration, levitate at slightly lower heights than control RBCs. The height difference, although observable when using bright field microscopy (20× and above using Kohler illumination), is not significant enough for observation via smartphone imaging in order to generate reliable measurements. Therefore, in order to improve reliability of the diagnostic test, increase the sensitivity and specificity of the approach, and minimize the assay time, dehydration was induced in RBC samples, amplifying the difference between control and SS RBC levitation. These results indicate an observable and statistically significant difference in the levitation of RBCs from people with sickle cell disease compared to control samples.

## Discussion and Conclusions

We demonstrated a digital and lightweight sickle cell disease identification platform which employs permanent magnets, a disposable microcapillary, optical components, and a Samsung Galaxy S4 with a custom-developed Android application. This strategy does not require antibodies or microscopy for diagnosis, and the resolution obtained with the built-in smartphone camera is sufficient to observe the difference in levitation height of SS cells compared to control RBCs under deoxygenated conditions. Though the procedure does require mixing with sodium metabisulfite and gadolinium, the amounts required are very small and do not present an unaffordable cost. The gadolinium used here is commercially available at a cost of $17.43 per 2 mL vial, which is sufficient for ~4,000 assays due to the small volume requirements of this procedure. An alternative material, gadolinium powder, is commercially available for less than $60/10 grams and can provide a lower cost solution to perform the same assays, considering that the gadolinium solution mixed with blood samples following collection does not need to meet FDA requirements for human use (as the Gadavist solution used here does). Furthermore, the proportionate quantity of sodium metabisulfite costs ~$0.01, which is sufficient for ~4,000 assays.

Though the sample mixing is performed via pipette in a laboratory setting, such sample preparation may be simplified for ease of use by untrained workers by finger-powered microfluidic mixing devices, such as the “squeeze-chip”^33^, which is capable of quantitative mixing of two fluids (such as blood and suspending medium). The remainder of the assay requires loading of the mixed sample into a microcapillary by capillary action, insertion into the apparatus, and imaging through the user-friendly smartphone application. These steps require minimal training and are performed in under a minute. Levitation of RBCs requires a 10 minute waiting period, but requires no user input during this time. It is important to note a limitation of these validation studies to only people who have been clinically diagnosed with sickle cell anemia (SS genotype), excluding people who only carry the sickle cell trait (i.e. those who are heterozygous for the sickle cell gene, AS genotype). Further testing with samples from heterozygous subjects will determine whether the magnetic levitation platform is capable of distinguishing between AS, SS, and control blood samples.

Screening and early diagnosis of sickle cell disease in newborns will ultimately allow proper care to be administered and prevent complications early on. Life-threatening complications of sickle cell disease include bacterial sepsis and stroke, particularly in children. Strokes in people living with sickle cell disease are thought to be caused by either adherence of sickle cells to the vascular walls or by lysis of the RBCs, which in turn causes activation of endothelial cells, coagulation, and constriction of blood vessels, occluding blood flow^34^. The incidence of stroke has been drastically reduced in those with sickle cell anemia through stroke risk screening with transcranial Doppler ultrasound and chronic prophylactic transfusion therapy^35^ Many strokes are “silent” strokes, which do not present the typical noticeable symptoms such as movement difficulties, affecting 1/4 of children with sickle cell disease by the age of 6 and 1/3 by the age of 14^36^. Though they often go undetected, but can result in significant morbidity and long-term damage to intellectual ability, academic ability, and memory. Silent strokes are generally only detectable through imaging such as an MRI. Thus, the most effective way to prevent such complications is to screen children for sickle cell disease in order to allow preventative measures to be taken. This smartphone based magnetic levitation platform, combined with the user friendly cell phone application, may be valuable for early screening of sickle cell disease with potential to be extended to other blood diseases.

## Methods

### Design and fabrication of the setup via 3D printer

The Sickle Cell Tester smartphone apparatus was designed in TinkerCAD, an online application for computer aided design (Autodesk, Inc., San Rafael, CA) (Supplementary Figure 1). The apparatus was printed with a Form 1+ stereolithography 3D printer (Formlabs, Somerville, MA). The main component is designed around a Samsung Galaxy S4 smartphone (dimensions 136.6 mm length by 69.8 mm width by 7.9 mm depth) such that the smartphone slides in lengthwise and is held securely upright along its length. Two N52-grade nickel plated NdFeB magnets with dimensions 50.8 mm length by 2 mm width by 5 mm thickness which are magnetized through the 5 mm thickness (custom design, K&J Magnetics, Inc., Pipersville, PA) are fixed with the same poles facing each other 1 mm apart. An aspheric lens with diameter 6.33 mm and numerical aperture 0.64 (Edmund Optics, Barrington, NJ) is used to achieve magnification of the sample; it is secured in a holder and placed between the built-in camera of the smartphone and the sample. The sample is illuminated on the opposite side from the camera using an LED connected in series to a standard slide switch and a CR2032 3 V battery with solid hookup wire and solder. The switch is fixed to the apparatus with adhesive and the battery is held with a battery holder.

### Ethical statement. Human Control RBC and SS Samples

The discarded de-identified samples were collected on a broad protocol for samples through the Sickle Cell Program at Boston Children’s Hospital, from patients with hemolytic anemia (including sickle) that was initially intended for evaluation of complement activation. An informed consent was given by all participants. All experiments involving human blood samples were performed in accordance with relevant Harvard Medical School and the University of Connecticut guidelines and regulations, and were approved by Harvard Medical School and the University of Connecticut. BCH Protocol # (X08-05-0255).

### Magnetic Levitation of RBCs

The blood samples are mixed with a Gadolinium-based (Gd^+^) paramagnetic medium, Gadavist (Bayer, Whippany NJ 07981) with 10 mM sodium metabisulfite (Sigma, St. Louis, MO) dissolved in Hank’s Balanced Salt Solution (55021C, Sigma, St. Louis, MI). The solution is drawn into a 0.98 mm by 0.98 mm square disposable microcapillary with 0.14 mm walls (8270–50, Vitrocom, Mountain Glass, NJ) by the capillary effect. The ends of the capillary tube are sealed with Critoseal to prevent leakage (215003, Leica Biosystems St. Louis, LLC, St. Louis, MO). Then, the sample is slid into the 1 mm space between the magnets and allowed to equilibrate for 10 minutes.

To test the effect of the gadolinium concentration on levitation, RBCs or polymer microspheres (used as a controlled-density micro-object to represent RBCs), were suspended in 10 mM sodium metabisulfite solution with varying concentrations of gadolinium. The solution was loaded into the square capillary tube and inserted into the Sickle Cell Tester. Time lapse images were taken periodically (time = 0 s corresponds to sample loading) and analyzed when the sample reached equilibrium (10 minutes). Results were averaged over 6 trails.

To compare the levitation of SS and control RBCs, cells were identified as local pixel intensity maxima. The width of confinement at equilibrium was calculated as the standard deviation of the vertical heights of the set of maxima in each image; each result is an average over 6 trials. Four control and eleven SS samples were tested and compared using a non-parametric Mann-Whitney-Wilcoxon two-sided test with a t approximation (α = 0.05).

### Cell phone application

The image analysis is performed using a custom-developed Android smartphone application which was developed in Eclipse Platform, an Android Developer Tool (The Android Open Source Project, developer.android.com). The application first averages the pixel intensities in the x-direction and creates an array of x-axis averages indexed by the y-axis location. The algorithm then measures the change in pixel intensity from pixel to pixel in the x-direction and creates an array of x-axis gradient data, also indexed by the y-axis location. The region of micro-object confinement is then identified as the peak in both the density and gradient arrays. A Guassian distribution fit is calculated for the pixel intensity array and displayed in the user interface. The mean and four times the standard deviation of the Gaussian distribution (in terms of y-axis pixels) is then calculated and reported to the user as ‘mean’ and ‘std’.

### Mathematical modeling of magnetic field distribution

The magnetic field, B, follows:


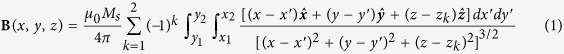


where *μ*_0_ is permeability of free space (=1.257 × 10^−6^ N/A^2^), *M*_*s*_ is the saturation magnetization of magnet (=6.36 × 10^5^ A/m), (x_1_, x_2_), (y_1_, y_2_), (z_1_, z_2_) denote the positions of the magnets with respect to x-, y-, and z-axes. The details of these derivations are have been reported previously^37^,^38^ and discussed in several other articles^39^,^40^,^41^.

### The x-component

B_x_ follows from Eq. (1)


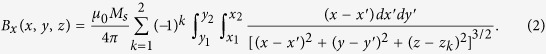


Integration with respect to x’ gives


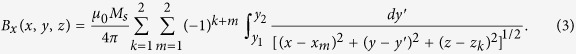


The remaining y’ integration can be evaluated by making a change of variable to α = y − y’. The resulting field expression is


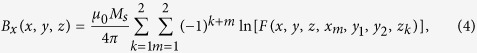



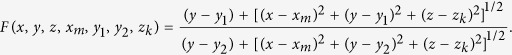


### The y-component

B_y_ also follows from Eq. (1),


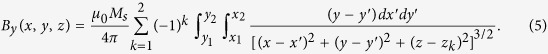


Integration with respect to y’ gives


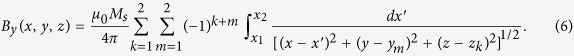


The remaining x’ integration is evaluated using a change of variable α = x − x’. The resulting field expression is


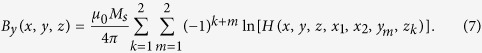



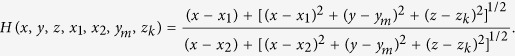


### The z-component

B_z_ is given by


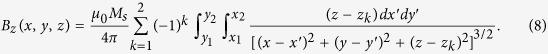


The x’ integration is performed using a change of variable α = x − x’


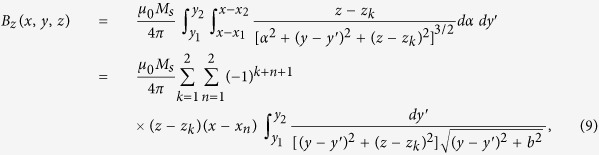


where b^2^ = (x − x_n_)^2^ + (z − z_k_)^2^. The remaining y’ integration is performed using a change of variable γ = y − y’. This gives


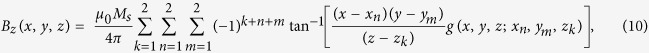


Here, equations 4, 7, and 10 were solved to plot the magnetic field distribution (Fig. 2a,b). Cells will be driven toward regions of minimal magnetic flux density (along the centerline between the two magnets) (Fig. 2c,d) and can be spatially confined in 3D or 2D magnetic traps.

## Additional Information

**How to cite this article**: Knowlton, S. M. *et al.* Sickle cell detection using a smartphone. *Sci. Rep.* **5**, 15022; doi: 10.1038/srep15022 (2015).

## Supplementary Material

Supplementary Information
