# Supplementary material for: Sickle cell detection using a smartphone
Source: Sci Rep. 2015 Oct 22;5:15022. doi: 10.1038/srep15022 (PMC4615037; doi:10.1038/srep15022)
Supplement: Supplementary Information [file srep15022-s1.pdf]

SUPPLEMENTARY FILE

Sickle cell detection using a smart phone

S.M. Knowlton[1], I. Sencan[2], Y. Aytar[3], J. Khoory[4], M.M. Heeney[5], I.C. Ghiran[4,a], and S. Tasoglu[1,6,a,¥]

[1]Department of Biomedical Engineering, University of Connecticut, 260 Glenbrook Road, Storrs, CT 06269.
[2]Department of Diagnostic Radiology, Yale School of Medicine, New Haven, CT 06520-8043.
[3]Computer Science and Artificial Intelligence Laboratory, Massachusetts Institute of Technology, Vassar Street Cambridge, MA 02139.
[4]Department of Medicine, Beth Israel Deaconess Medical Center, Harvard Medical School, Boston MA 02115.
[5]Sickle Cell Program, Pediatric Blood Disorders Center, Boston Children's Hospital, Harvard Medical School, Boston MA 02115.
[6]Department of Mechanical Engineering, University of Connecticut, Storrs, CT 06269.

[a]These authors equally contributed to this study.
[¥]Corresponding Author: Savas Tasoglu (savas@engr.uconn.edu)

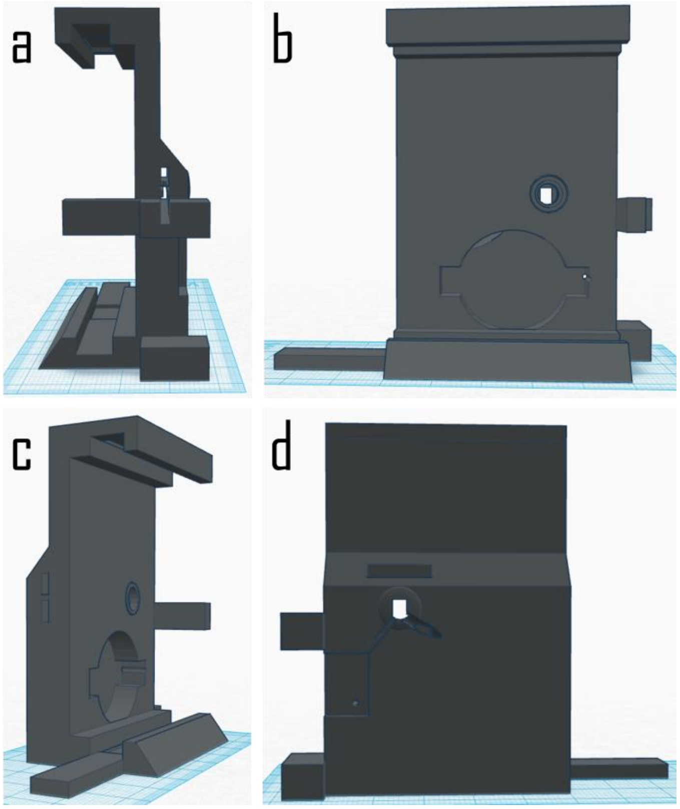

Supplementary Figure 1. Images of the 3D designed Sickle Cell Tester.

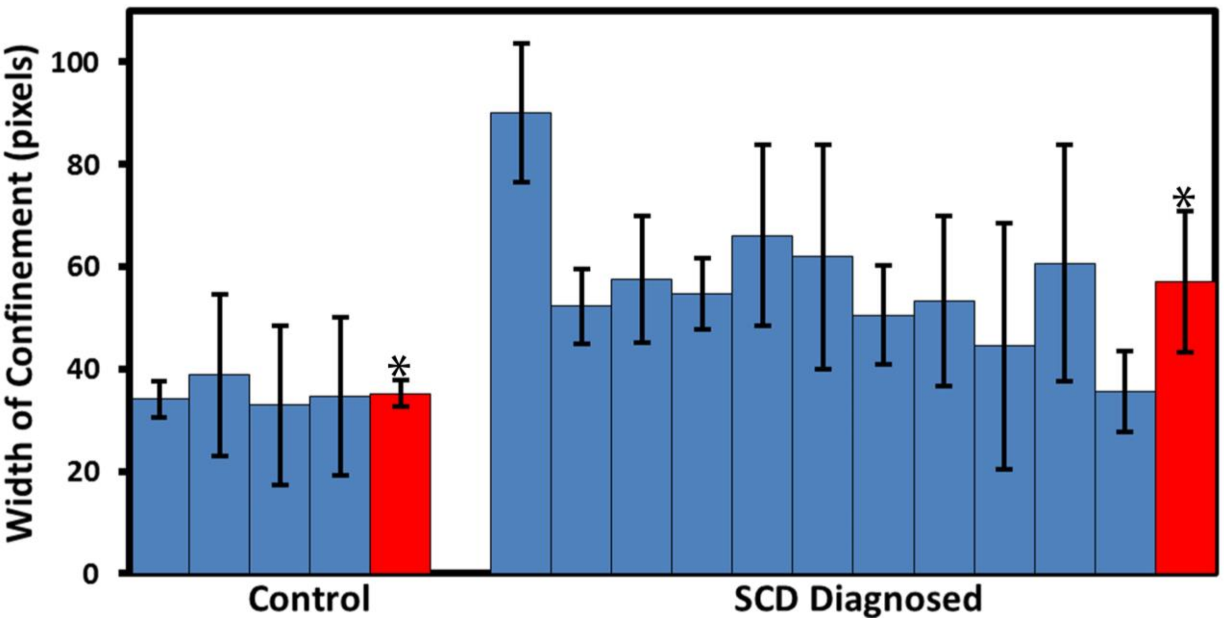

Supplementary Figure 2. Confinement width of 4 control and 11 SS RBC samples as measured by the standard deviation of the RBC levitation heights. Blue bars each represent the average of 6 trials for each blood sample tested with standard deviation bars. Red bars represent the average across all control and all sickle cell samples tested with standard deviation bars showing variation within the samples.

Methods

Imaging procedure and the optical setup:

The sample is illuminated by a white LED driven with a 3V CR2032 coin battery. A 120-grit ground glass diffuser (DG05-120, Thorlabs, Inc, Newtown, NJ) between the LED and capillary tube ensures uniformity of illumination across the imaging field of view (FOV). Objects within this FOV are imaged onto the active area of the cellphone camera (Sony IMX135, 1.12μm pixel pitch, 4208x3120 pixels) by using a 4-f imaging system. This 4-f imaging system is formed by using the built-in lens unit of the cellphone camera with an effective focal length (EFL) of 4.2mm and an external aspheric lens with diameter 6.33 mm, numerical aperture 0.64 mm, and effective focal length 4.03 mm (87-161, Edmund Optics, Barrington, NJ). The spacing between the camera lens and the external lens is adjusted as the summation of their focal lengths (~8.23 mm). Also, the ratio between these focal lengths provides approximately unit magnification (M = 4.2/4.03 = ~1.04), and this ratio sets the effective pixel size of the imaging optics on the object plane as ~1.17μm. Working with this effective pixel size provides a sufficient sampling rate for reliable localization of objects and does not reduce the FOV, which spans entire depth of the capillary tube (1 mm) without aberrations. The alignment of the imaging system was experimentally optimized to maximize the resolution and minimize optical aberrations (Pincushion distortions, chromatic aberrations, and vignetting). Images were captured using the stock camera application that comes standard with the Samsung Galaxy S4. Autofocus was used to focus images by manually selecting the region of confinement.
